# Seroprevalence of Zika and Dengue Virus Antibodies among Migrant Workers, Taiwan, 2017

**DOI:** 10.3201/eid2504.181449

**Published:** 2019-04

**Authors:** Guey Chuen Perng, Tzu-Chuan Ho, Hsin-I Shih, Chia-Hua Lee, Pei-Wen Huang, Chih-Huan Chung, Nai-Ying Ko, Wen-Chien Ko, Yu-Wen Chien

**Affiliations:** National Cheng Kung University College of Medicine, Tainan, Taiwan (G.C. Perng, T.-C. Ho, H.-I. Shih, C.-H. Lee, P.-W. Huang, N.-Y. Ko, W.-C. Ko, Y.-W. Chien);; National Cheng Kung University Hospital, Tainan (H.-I. Shih, N.-Y. Ko, W.-C. Ko, Y.-W. Chien);; Kuo General Hospital, Tainan (C.-H. Chung)

**Keywords:** Zika virus, dengue virus, dengue, seroprevalence, migrant workers, Southeast Asia, neutralization tests, Flavivirus, viruses, Taiwan

## Abstract

A serosurvey of 600 workers newly arrived in Taiwan from 4 Southeast Asia countries showed that 18 (3%) were positive for Zika virus IgM; 6 (1%) fulfilled the World Health Organization criteria for laboratory-confirmed recent Zika virus infection. The incidence of Zika virus infection in Southeast Asia might be underestimated.

Approximately 690,000 migrant workers live in Taiwan; most are from 4 countries in Southeast Asia: Indonesia, the Philippines, Thailand, and Vietnam. Although these migrants are valuable to the workforce, they may also bring the risk of spreading mosquitoborne diseases. Most imported cases of Zika virus and dengue virus (DENV) infections in Taiwan come from Southeast Asia. Although screening for fever at international airports was implemented in Taiwan in 2003 (*1*), most Zika virus and DENV infections are inapparent and cannot be detected.

For assessing the true disease burden of flavivirus infections, seroprevalence studies are effective. Recently, several serosurveys of Zika virus infections were conducted in Africa, Oceania, Latin America, and the Caribbean (*2*). Although small outbreaks of Zika virus infection have been reported in Singapore, Vietnam, and Thailand (*3*–*5*), seroprevalence in Southeast Asia countries remains largely unknown. To estimate the incidence of Zika virus and DENV infections in Southeast Asia and to evaluate the risk of importing these viruses into Taiwan, we investigated seroprevalence of IgM and IgG against these viruses among newly arrived migrant workers in Taiwan.

## The Study

Migrant workers are required by law to undergo preemployment health examinations within 3 days of arrival in Taiwan. For this study, we recruited 600 newly arrived migrant workers from Indonesia, the Philippines, Thailand, and Vietnam (150 workers from each country) who received preemployment examinations at a regional hospital in Tainan, Taiwan, during June–August 2017. Workers who were >20 years of age and willing to participate were eligible without specific exclusion criteria. We used commercial ELISAs (https://focusdx.com) to test for IgM and IgG against dengue virus, anti-Zika virus IgG ELISA (Euroimmun AG, https://www.euroimmun.com) to test for IgG against Zika virus, and InBios Zika Detect IgM Capture ELISA (http://www.inbios.com) to test for IgM against Zika virus (because this assay seems to have higher sensitivity) (*6*). All tests were performed and interpreted according to manufacturers’ instructions. We performed plaque reduction neutralization tests (PRNTs) on a subgroup of samples to detect neutralizing antibodies against 4 DENV serotypes (DENV-1, strain Hawaii; DENV-2, strain 16681; DENV-3, strain H87; DENV-4, strain H241) and 2 Zika virus strains (strain MR766 and 1 clinical isolate from a patient who acquired infection in Thailand). We calculated titers required to reduce dengue and Zika viral plaques by 50% (PRNT_50_) and 90% (PRNT_90_) (Appendix). For persons positive for Zika virus IgM, we used the interim case definition of laboratory-confirmed cases of recent Zika virus infection defined by the World Health Organization to see whether they fulfilled these criteria: IgM antibody against ZIKV positive and PRNT_90_ for ZIKV with titer ≥20 and ZIKV PRNT_90_ titer ratio ≥4 compared to other flaviviruses (*7*). This study was approved by the institutional review board of National Cheng Kung University Hospital (approval no. A-ER-106-045).

Most migrant workers were young adults 20–39 years of age (Appendix Table 1). Of the 600 workers, 18 (3.0%) were positive for Zika virus IgM and 233 (38.8%) were positive for Zika virus IgG. Only 3 (0.5%) workers had detectable DENV IgM, but 484 (80.7%) had DENV IgG. Seroprevalence of IgG against Zika virus and DENV was much lower in Vietnam than in the other 3 countries (Appendix Tables 2, 3).

Among all workers, 227 (37.8%) had IgG against both Zika virus and DENV, 6 (1.0%) had only Zika virus IgG, 257 (42.8%) had only DENV IgG, and 110 (18.3%) were negative for both (Table 1). All 18 workers positive for Zika virus IgM were negative for DENV IgM, indicating that false-positive results from cross-reactivity with DENV IgM or polyclonal B cell stimulation were unlikely. We found that 6 (1%) workers positive for Zika virus IgM had Zika virus PRNT_90_ titers >20, and their Zika virus PRNT_90_ titer ratio was ≥4 compared with that of 4 serotypes of DENV (Table 2; Appendix Table 4). Although we did not perform PRNT for flaviviruses other than Zika virus and DENV, we assumed that these 6 workers fulfilled the World Health Organization criteria of confirmed Zika virus infection. All 3 workers with positive DENV IgM had detectable PRNT_50_ and PRNT_90_ titers against single or multiple DENV serotypes; thus, the positive ELISA results for DENV IgM were considered true positives. Among 6 participants with positive Zika virus IgG but negative DENV IgG, 5 had a high PRNT_50_ titer against Zika virus (Appendix Table 4).

**Table 1:** Zika virus and DENV serostatus for 600 migrant workers from Southeast Asia, Taiwan, 2017*

Serostatus	No. (%) workers					
	Zika virus IgG+			Zika virus IgG–		Total
	Zika virus IgM+	Zika virus IgM–		Zika virus IgM+	Zika virus IgM–	
DENV IgG+						
DENV IgM+	0	3 (0.5)		0	0	3 (0.5)
DENV IgM–	11 (1.8)	213 (35.5)		6 (1.0)	251 (41.8)	481 (80.2)
DENV IgG–						
DENV IgM+	0	0		0	0	0
DENV IgM–	0	6 (1.0)		1 (0.2)	109 (18.2)	116 (19.3)
Total	11 (1.8)	222 (37.0)		7 (1.2)	360 (60.0)	600 (100)

*DENV, dengue virus; +, positive; –, negative.

**Table 2:** Serostatus and neutralizing antibody titers for Zika virus and DENV among 11 migrant workers from Southeast Asia who were IgM and IgG positive for Zika virus, Taiwan, 2017*

Worker no., age, y/sex	ELISA										
	Zika virus IgM	Zika virus IgG	DENV IgM	DENV IgG		90% PRNT titer					
						DENV-1	DENV-2	DENV-3	DENV-4	Thai	MR766
ID01, 21/F†	+	+	–	+		10	10	<10	<10	149	399
ID02, 37/F	+	+	–	+		609	1,401	40	<10	10	<10
ID03, 28/M	+	+	–	+		138	505	11	149	438	<10
VN01, 34/M†	+	+	–	+		<10	40	<10	<10	40	160
PH01, 28/F†	+	+	–	+		124	440	147	<10	1486	>2,560
PH02, 24/M†	+	+	–	+		<10	10	<10	<10	227	639
PH03, 24/F†	+	+	–	+		<10	<10	<10	<10	800	1279
TH01, 42/M	+	+	–	+		<10	40	<10	<10	107	143
TH02, 46/M†	+	+	–	+		<10	513	<10	<10	593	>2,560
TH03, 43/F	+	+	–	+		405	310	40	40	10	1,215
TH04, 31/M	+	+	–	+		>2,560	1,360	1,600	10	1,468	1,599

*DENV, dengue virus; DENV-1–4, DENV serotypes 1–4; MR766, African Zika virus strain MR766; PRNT, plaque reduction neutralization test; Thai, 1 Zika virus isolate from a worker with an imported case acquired in Thailand. †Six persons positive for Zika virus IgM fulfilled the criteria for laboratory confirmation of recent Zika virus infection adopted with definition according to the World Health Organization (*7*).

## Conclusions

Zika virus IgM persists for ≈12 weeks (*8*); therefore, our results suggest that 1% of the workers had confirmed Zika virus infection within 3 months before blood collection, implying that the incidence of Zika virus infection in Southeast Asia might be severely underestimated. The median duration of viremia is 2 weeks (*9*); thus, some workers might have entered Taiwan during their viremia period and had the potential to spread Zika virus through *Aedes* mosquitoes in Taiwan. In addition, Zika virus can be detected in semen up to 6 months after symptom onset (*10*); thus, Zika virus transmission through sexual contact with these workers, who are at a sexually active age, is also possible. Furthermore, 3 female workers with confirmed Zika virus infection were of childbearing age, raising concerns about the risk for congenital infection.

The infectiousness of persons with asymptomatic Zika virus infection remains unknown. However, a recent study of DENV showed that asymptomatic persons could infect mosquitoes despite their lower average level of viremia (*11*). A recent modeling analysis estimated that 84% of DENV transmission is attributable to persons with inapparent infections because these persons are more mobile (*12*). If the infection characteristics of Zika virus are similar to those of DENV, the ability of fever screening programs at international airports and ports to prevent importation of Zika virus from migrant workers and travelers will be limited.

In this study, we may have overestimated Zika virus IgG seroprevalence (38.8%) because of false positivity resulting from cross-reactivity; nevertheless, the observed seroprevalence is comparable with that in Martinique (42.2%) and French Polynesia (49%) but lower than that in Brazil (63.3%) and Micronesia (73%) (*2*). It remains unclear why only very few cases of Zika virus–related microcephaly have been reported in Southeast Asia (*13*) despite such high seroprevalence. Possible explanations are differences in virus strains, differences in host factors, and limitations of the surveillance system (*14*).

To our surprise, seroprevalence of DENV IgM was lower than that of Zika virus IgM. In DENV-hyperendemic countries, children may have been exposed to multiple DENV serotypes and then acquired immunity; therefore, the incidence of dengue in adults is relatively low. Also of note, we observed much lower seroprevalences of Zika virus and DENV among workers in Taiwan from Vietnam, which may be because most of these workers originally came from rural areas in subtropical northern Vietnam, where the population density and climate are not suitable for establishing endemic transmission cycles of mosquitoborne viruses (*15*).

Our finding that 1% of migrant workers from Southeast Asia had laboratory-confirmed recent Zika virus infection suggests that the incidence of Zika virus infection in this region is underestimated. Given the convenience of flight for global travel, the risk for international dissemination of Zika virus by workers and travelers originating from Southeast Asia cannot be neglected.

Appendix Plaque reduction neutralization test methods and additional results for study of seroprevalence of Zika and dengue virus infections among migrant workers, Taiwan, 2017.
