# Supplementary material for: Seroprevalence of Zika and Dengue Virus Antibodies among Migrant Workers, Taiwan, 2017
Source: Emerg Infect Dis. 2019 Apr;25(4):814–6. doi: 10.3201/eid2504.181449 (PMC6433042; doi:10.3201/eid2504.181449)
Supplement: Appendix — Plaque reduction neutralization test methods and additional results for study of seroprevalence of Zika and dengue virus infections among migrant workers, Taiwan, 2017. [file 18-1449-Techapp-s1.pdf]

Article DOI: https://doi.org/10.3201/eid2504.181449

Seroprevalence of Zika and Dengue Virus Antibodies among Migrant Workers, Taiwan

Appendix

Plaque Reduction Neutralization Test for Dengue Virus and Zika Virus

Plaque reduction neutralization tests (PRNT) were performed to detect the antibody neutralization titers for four serotypes of dengue virus (DENV1 – 4) and two strains of Zika virus (ZIKV). The viruses used in current PRNT tests were DENV1 (strain Hawaii), DENV2 (strain 16681), DENV3 (strain H87), and DENV4 (strain H241), and ZIKV (strain MR766 and one recent clinical isolate from an imported subject who was infected in Thailand). The PRNT protocols for DENV and ZIKV were previously described (*1*,*2*). Briefly, PRNTs were performed in baby hamster kidney fibroblasts (BHK-21) cells and Vero cells for DENV and ZIKV, respectively. A total of 7×10^5 cell per well of BHK-21 cells and Vero cells were seeded in 6-well plates 1 day before PRNT assays. The complement in human serum was inactivated at 56°C for 30 minutes. The inactivated serum was then subjected to 4-fold serial dilution from 1:10 to 1:2560 with 2% DMEM. Viruses at 10^4 PFU in 250uL were mixed with equal volume of diluted serum at 37°C for 30 minutes. The virus-serum mixtures were agitated every 15 minutes, and then 400uL of mixtures were added into each well of 6-well plates that had been seeded with cells. The plates were incubated at 37°C in 5% CO2 incubator for 2 hours, the inoculants were then aspirated, and overlaid with 3mL per well of 1% methyl cellulose solutions (2% FBS of DMEM, pH7.4~7.6). The plates were incubated at 37°C in 5% CO_2 incubator for 7 days. The overlay media were removed from plates after 7 days, and the cells were stained with 2mL of crystal violet for 2 hours and followed by rinsing with water. Plaque numbers were counted by the naked eye under the transmitted light box. Antibody titers were calculated based on reciprocal of the diluted serum titers, in which 50% and 90% reduction in plaque counts ($PRNT_{50}$ and $PRNT_{90}$) compared to controls were obtained by using GraphPad Prism version 7 for Windows (GraphPad Software, La Jolla, CA, USA). If the titer could not be calculated, it was

expressed as the reciprocal of the highest diluted serum titers by showing ≥50% or ≥90% reduction in plaque counts as compared with that of controls.

References

1. Morens DM, Halstead SB, Repik PM, Putvatana R, Raybourne N. Simplified plaque reduction neutralization assay for dengue viruses by semimicro methods in BHK-21 cells: comparison of the BHK suspension test with standard plaque reduction neutralization. J Clin Microbiol. 1985;22:250–4. PubMed

2. Müller JA, Harms M, Schubert A, Mayer B, Jansen S, Herbeuval JP, et al. Development of a high-throughput colorimetric Zika virus infection assay. Med Microbiol Immunol (Berl). 2017;206:175–85. PubMed http://dx.doi.org/10.1007/s00430-017-0493-2

Appendix Table 1. Sex and age distribution of migrant workers from four Southeast Asian countries.

	N (% of total)				
Category	Indonesia	Philippines	Thailand	Vietnam	All
Total	150	150	150	150	600
Sex					
Female	75 (50.0%)	129 (86.0%)	30 (20.0%)	63 (42.0%)	297 (49.5%)
Male	75 (50.0%)	21 (14.0%)	120 (80.0%)	87 (58.0%)	303 (50.5%)
Age					
20–29	66 (44.0%)	120 (80.0%)	52 (34.7%)	83 (55.3%)	321 (53.5%)
30–39	73 (48.7%)	29 (19.3%)	54 (36.0%)	57 (38.0%)	213 (35.5%)
≥40	11 (7.4%)	1 (0.7%)	44 (29.3%)	10 (6.7%)	66 (22%)

Appendix Table 2. Seroprevalence of Zika virus-specific IgG and IgM among migrant workers from four Southeast Asian countries.

	No. of positive (% positive)									
	Indonesia		Philippines		Thailand		Vietnam		All	
Category	IgM	IgG	IgM	IgG	IgM	IgG	IgM	IgG	IgM	IgG
Total	7 (4.7)	65 (43.3)	5 (3.3)	83 (55.3)	5 (3.3)	76 (50.7)	1 (0.7)	9 (6.0)	18 (3.0)	233 (38.8)
Sex										
Female	4 (5.3)	27 (36.0)	4 (3.1)	71 (55.0)	1 (3.3)	19 (63.3)	0 (0.0)	2 (3.2)	9 (3.0)	119 (40.1)
Male	3 (4.0)	38 (50.7)	1 (4.8)	12 (57.1)	4 (3.3)	57 (47.5)	1 (1.1)	7 (8.0)	9 (3.0)	114 (37.6)
Age										
20–29	2 (3.0)	31 (47.0)	5 (4.2)	68 (56.7)	0 (0.0)	22 (42.3)	0 (0.0)	6 (7.2)	7 (2.2)	127 (39.6)
30–39	5 (6.8)	30 (41.1)	0 (0.0)	15 (51.7)	1 (1.9)	31 (57.4)	1 (1.8)	3 (5.3)	7 (3.3)	79 (37.1)
≥40	0 (0.0)	4 (40.0)	0 (0.0)	0 (0.0)	4 (9.8)	23 (52.3)	0 (0.0)	0 (0.0)	4 (6.1)	27 (40.9)

Appendix Table 3. Seroprevalence of dengue virus-specific IgG and IgM among migrant workers from four Southeast Asian countries.

	No. of positive (% positive)									
	Indonesia		Philippines		Thailand		Vietnam		All	
Category	IgM	IgG	IgM	IgG	IgM	IgG	IgM	IgG	IgM	IgG
Total	0(0.0)	136 (90.7)	1 (0.7)	142 (94.7)	2 (1.3)	136 (90.7)	0 (0.0)	70 (46.7)	3 (0.5)	484 (80.7)
Sex										
Female	0 (0.0)	67 (89.3)	1 (0.8)	122 (94.6)	0 (0.0)	26 (86.7)	0 (0.0)	26 (41.3)	1 (0.3)	241 (81.1)
Male	0 (0.0)	69 (92.0)	0 (0.0)	20 (95.2)	2 (1.7)	110 (91.7)	0 (0.0)	44 (50.6)	2 (0.7)	243 (80.2)
Age										
20–29	0 (0.0)	61 (92.4)	1 (0.8)	115 (95.8)	0 (0.0)	42 (80.8)	0 (0.0)	32 (38.6)	1 (0.3)	250 (77.9)
30–39	0 (0.0)	66 (90.4)	0 (0.0)	26 (89.7)	2 (3.7)	52 (96.3)	0 (0.0)	33 (57.9)	2 (0.9)	177 (83.1)
≥40	0 (0.0)	9 (81.8)	0 (0.0)	1 (100.0)	0 (0.0)	42 (95.5)	0 (0.0)	5 (50.0)	0 (0.0)	57 (86.4)

Appendix Table 4. Serostatuses and titers of neutralizing antibodies for Zika virus and dengue virus infection among selected workers*

			ELISA				$PRNT_{90}$						$PRNT_{50}$					
Worker No.	Age	Sex	Zika IgM	Zika IgG	DEN IgM	DEN IgG	D1	D2	D3	D4	Thai	MR766	D1	D2	D3	D4	Thai	MR766
ID01†	21	F	+	+	-	+	10	10	<10	<10	149	399	78	40	36	33	150	400
ID02	37	F	+	+	-	+	609	1401	40	<10	10	<10	610	1402	640	39	102	38
ID03	28	M	+	+	-	+	138	505	11	149	438	<10	139	507	68	150	440	<10
VN01†	34	M	+	+	-	+	<10	40	<10	<10	40	160	<10	153	<10	<10	253	1066
PH01†	28	F	+	+	-	+	124	440	147	<10	1486	>2560	126	441	149	10	>2560	>2560
PH02†	24	M	+	+	-	+	<10	10	<10	<10	227	639	<10	259	40	<10	160	640
PH03†	24	F	+	+	-	+	<10	<10	<10	<10	800	1279	<10	25	<10	<10	>2560	>2560
TH01	42	M	+	+	-	+	<10	40	<10	<10	107	143	<10	214	<10	35	109	160
TH02†	46	M	+	+	-	+	<10	513	<10	<10	593	>2560	38	515	31	38	640	>2560
TH03	43	F	+	+	-	+	405	310	40	40	10	1215	406	312	249	269	764	>2560
TH04	31	M	+	+	-	+	>2560	1360	1600	10	1468	1599	>2560	>2560	>2560	82	>2560	1600
ID04	31	F	+	-	-	+	<10	10	<10	<10	<10	<10	<10	40	<10	<10	<10	82
ID05	37	M	+	-	-	+	<10	10	40	<10	<10	<10	<10	40	40	35	<10	459
ID06	32	M	+	-	-	+	<10	10	34	<10	<10	40	<10	40	39	34	40	160
ID07	33	F	+	-	-	+	10	34	<10	<10	<10	<10	40	39	38	<10	<10	38
PH04	23	F	+	-	-	+	<10	40	<10	10	<10	29	<10	160	<10	40	<10	30
TH05	43	M	+	-	-	+	<10	92	141	<10	10	155	38	93	143	<10	101	>2560
PH05	22	F	-	+	+	+	<10	<10	10	10	10	579	<10	37	40	40	239	589
TH06	33	M	-	+	+	+	10	160	<10	<10	<10	<10	62	1102	28	38	40	776
TH07	37	M	-	+	+	+	<10	446	<10	513	<10	<10	10	448	<10	640	<10	<40
VN02	23	F	-	+	-	-	<10	<10	<10	<10	<10	<10	<10	<10	<10	<10	111	<10
PH06	33	M	-	+	-	-	<10	<10	<10	<10	144	40	<10	<10	<10	<10	145	640
TH08	48	M	-	+	-	-	<10	382	<10	<10	<10	356	<40	384	40	36	107	357
TH09	41	M	-	+	-	-	<10	40	<10	<10	<10	40	36	337	<10	<10	108	80
TH10	21	M	-	+	-	-	<10	<10	<10	<10	<10	<10	<10	<10	<10	<10	374	<10
TH11	30	M	-	+	-	-	<10	<10	<10	<10	<10	<10	<10	<10	10	<10	<10	10

*ELISA, enzyme-linked immunosorbent assay; PRNT, plaque reduction neutralization test; ZIKV, Zika virus; DENV, dengue virus; D1, dengue virus serotype 1; D2, dengue virus serotype 2; D3, dengue virus serotype 3; D4, dengue virus serotype 4; Thai, one Zika virus isolate from an imported cases who got infection in Thailand; MR766, the African Zika virus strain MR766.

†Six people with positive anti-ZIKV IgM fulfilled the criteria for laboratory confirmation of recent ZIKV infection.
